# The role of uropathogenic *Escherichia coli* adhesive molecules in inflammatory response- comparative study on immunocompetent hosts and kidney recipients

**DOI:** 10.1371/journal.pone.0268243

**Published:** 2022-05-23

**Authors:** Bartosz Wojciuk, Karolina Majewska, Bartłomiej Grygorcewicz, Żaneta Krukowska, Ewa Kwiatkowska, Kazimierz Ciechanowski, Barbara Dołęgowska

**Affiliations:** 1 Laboratory for Diagnostic Immunology, Chair of Microbiology, Immunology and Laboratory Medicine, Pomeranian Medical University in Szczecin, Szczecin, Poland; 2 Department of Laboratory Medicine, Chair of Microbiology, Immunology and Laboratory Medicine, Pomeranian Medical University in Szczecin, Szczecin, Poland; 3 Laboratory for Medical Microbiology, Chair of Microbiology, Immunology and Laboratory Medicine, Pomeranian Medical University in Szczecin, Szczecin, Poland; 4 Clinic of Internal Medicine, Nephrology and Transplantation, Pomeranian Medical University in Szczecin, Szczecin, Poland; CINVESTAV-IPN, MEXICO

## Abstract

**Background:**

Urinary tract infections (UTI) represent one of the most common contagious diseases in humans. Uropathogenic *Escherichia coli* (UPEC) strains are recognized as the most frequent causative agent, and these express a range of virulence factors including the adhesins. Immune response to UPEC under immunosuppression has not been fully understood yet. Interleukin 1β (IL1β), 6 (IL6) and 17 (IL17) represent clinically relevant markers of inflammation.

**Aim:**

The study aimed to investigate the interplay between UPEC genotype and hosts’ immune status in shaping local inflammatory response in the course of an UTI episode. The respective numbers of: 18 kidney recipients with UPEC UTI, 28 immunocompetent hosts with UPEC UTI and 29 healthy controls were involved. Urine IL1β, IL6, and IL17/creatinine ratios in relation to *fimH*, *csgA*, *papC*, *tosA*, and *flu* genes presence in UPEC isolated from the urine samples were analyzed. Apart from traditional statistics, also machine learning algorithms were applied.

**Results:**

The urine levels of IL1β and IL 6 were similar in kidney recipients and the immunocompetent hosts. IL1β levels were higher in both kidney recipients and immunocompetent hosts than in controls, while IL6 levels were higher only in immunocompetent hosts than in controls. In the machine learning classification model, high urine IL17 levels were significantly more prevalent in controls, while low IL17 levels in urines infected with Ag43-positive UPEC strains, regardless of the host’s immune status. In the traditional statistical analysis, IL17 levels appeared significantly higher in urine samples from kidney recipients infected with Ag43–negative UPEC strains.

**Conclusions:**

In the UTI- affected patients, the combination of the immune status of an individual and Ag43 status of the UPEC strain determined urine IL17 level in the analyzed group. However, IL17 levels above median were overall more prevalent in controls.

## Introduction

Urinary tract infections (UTI) are recognized as outstandingly common among the population [1]. They affect over 150 million individuals annually, representing respectively 40–50% of nosocomial and 10–20% of community-acquired infections in adults [2–5]. UTI constitute a significant challenge in post-kidney transplantation care as well. Together with latent viruses such as human cytomegalovirus (CMV) and BK virus (BKV) reactivations, these are indicated as the most frequent infectious complications in this group [6–8].

Uropathogenic *Escherichia coli* (*E*. *coli*) strains (UPEC) are also recognized as the most frequent UTI causative agent regardless of either nosocomial or the community-acquired character of the episode. It is also true for kidney recipients [6–8]. UPEC significantly contribute to the clinically relevant set of extraintestinal pathogenic *E*. *coli* strains together with neonatal meningitis *E*. *coli* (NMEC) and *sepsis-associated E*. *coli* (SEPEC). Despite being recovered from the intestinal microbiota of healthy individuals, numerous virulence factors make UPEC pathogenic in the unique urinary tract environment. The virulence repertoire of UPEC strains includes adhesive factors, toxins, iron uptake systems, and others [9, 10].

Adhesive molecules are considered to substantially shape the interaction between the host and UPEC during the UTI episode. These significantly contribute to the successful survival of the pathogen inside the urinary tract. It is mostly connected with a direct physical interaction between the planktonic and attached bacteria in biofilm and the host cells. It is also critical for long-term survival, drug-resistance, and eventually, UTI relapses. Furthermore, some of these are also recognized as ligands for innate immunity receptors [11, 12].

UPEC strains produce several groups of molecules, functionally identified as adhesins, such as fimbriae (type I fimbriae, P fimbriae, S fimbriae, Afa/Dr proteins, curli*)*, autotransporter adhesins, non-fimbrial adhesins [9, 10, 12].

Type I fimbriae encoded by the *fim* operon represent one of the most common virulence factors among UPEC. Due to their interaction with uroplakin 1a, these enable bacterial cells’ adherence to the urothelium. Besides, these ligate Toll-like receptor 4 (TLR4) and trigger the innate immune response. Similarly, to type I fimbriae, P fimbriae encoded by *pap* operon contribute to inflammation triggering *via* TLR4 ligation. P fimbriae adhere to urothelium glycolipids, and this further results in ceramide release. P fimbriae are also considered to be associated with upper urinary tract infections [11, 13, 14].

In contrast, *csg-*encoded curli express their affinity to the extracellular matrix and bind laminin and fibronectin and they also bind to TLR 2. It makes them a significant factor in biofilm formation [11].

Non-fimbrial adhesins such as Type one secretion A (tosA) are highly associated with urinary tract colonization and target mostly upper urinary tract epithelium. Their immunogenicity has also been proven [15].

Autotransporter proteins appear less described among virulence factors in UPEC. These can act differently, either as toxins, adhesins, or proteases. Due to their characteristic structure, once transported from the cytoplasm, these can either get attached to the bacterial surface or secreted outside the bacterial cell and act as toxins. Within this group, Ag43, encoded by the *flu* gene, is identified as an adhesin correlated with a strong biofilm-forming potential [11, 16, 17].

Local inflammatory reaction triggered by Gram-negative bacteria has been well recognized. This reaction is primarily dependent on innate immune cells, such as macrophages but also epithelial cells. These, when activated by lipopolysaccharide, secrete proinflammatory cytokines: interleukin 1 (IL1) and interleukin 6 (IL6). Consequently, these cytokines act locally and systemically [18–22]. Interleukin 17 (IL17), discovered over ten years ago, has been connoted with acute infections and autoimmune diseases. This cytokine is supposed to improve the chemotactic activity of neutrophils. While in autoimmune disorders, IL 17 is generated mainly by pathologically active Th 17 cells, in the urinary tract, it is synthesized by Tγδ lymphocytes, which can recognize bacterial ligands independently on MHC restriction [23]. However, the molecular patterns capable of triggering IL 17 production have not been fully identified yet. All three cytokines have been investigated in the context of kidney allograft injury [24–27].

Despite increasing knowledge about bacterial adhesins in general, still relatively little is known about their impact on immunity. Nevertheless this is outstandingly significant for the understanding of host-pathogen interactions where both- the host’s condition and the pathogen’s phenotype contribute to local conditions. Consequently, there is an emerging need for a personalized approach towards each individual [28, 29] and the complex analyses are essential to assess the hierarchy of certain coexisting factors in shaping the local inflammatory milieu. Machine learning algorithms, dedicated to this approach are primarily associated with big data sets. However, different techniques, including cross-validation protocol, e.g., make these accessible for smaller data samples as well. [30, 31].

This study aimed to investigate the quality of inflammatory response in the urine of kidney recipients and immunocompetent hosts concerning adhesive molecules genes present in UPEC strains isolated from urine samples, respectively.

## Material and methods

### Patients

A group of 75 individuals was included in the study. The group consisted of 28 immunocompetent patients (21 women, 7 men) admitted to the nephrology department between March 2019 and March 2020 with microbiologically confirmed UTI, 18 kidney recipients (14 women, 4 men, average 88 months post-transplant) with documented UTI episodes between March 2019 and March 2020 and 29 healthy controls (15 women, 14 men). The UTI episode was indicated with a clinical image (dysuria) combined with leucocyturia in the immunocompetent and clinical image, leucocyturia and/or decreased allograft function in kidney recipients. All enrolled patients had the UTI confirmed by urine culture with confirmed bacterial growth over 10 ^5^ colony forming units/ml. The details of bacterial identification have been described in section *Bacterial identification and virulence characteristics*. Healthy volunteers were questionaired according to current UTI symptoms, previous UTI history and concomitant diseases including urological disorders. The individuals with previous UTI episodes and coexisting UTI-predisposing conditions were excluded from the study. The average age in particular groups was respectively: immunocompetent hosts 68 SD 17, kidney recipients 51 SD 15, controls 35 SD 13. According to the MDRD formula, the average glomerular filtration rate (eGFR) in particular groups was respectively: immunocompetent hosts—48 ml/min SD 31, kidney recipients—41 ml/min SD 13, controls over 60 ml/min. During UTI diagnosis, 16 of the kidney recipients were treated with calcineurin inhibitors, 2 with m TOR inhibitors, 14 with mofetil mycophenolate (MMF), and 8 with glucocorticosteroids (GCS). Precise immunosuppression protocols are listed below:

1. Tacrolimus, mofetil mycophenolate—10 persons
2. Tacrolimus, mofetil mycophenolate, prednisone—4 persons
3. Tacrolimus, dephlasacort—1 person
4. Cyclosporine, prednisone—1 person
5. Everolimus, dephlasacort—1 person
6. Sirolimus, prednisone—1 person

### Urine samples collection

Voided urine collected for routine microbiological diagnostics was further centrifuged at 2000 rpm for 20 min and stored at -70°C until consecutive analyses. Voided urine was obtained from controls as well, proceeded, and stored analogously.

### Cytokines assessment

IL1β, IL6, and IL 17 levels were measured with Human IL1β ELISA Kit (cat. no. RAB0273-1KT), Human IL6 ELISA Kit (cat. no. RAB0306-1KT), and Human IL17 ELISA Kit (cat. no. RAB0262-1KT), respectively (Sigma Aldrich, Germany), according to the manufacturer’s guidelines. All kits have been certified as standardized for urine. Creatinine concentration was measured in each urine sample (Cobas 8000, c-502, Roche, USA). Interleukin/creatinine ratio was calculated for each urine sample and each interleukin, respectively.

### Bacterial identification and virulence characteristics

The number of thirty eight UPEC strains isolated from patients was analyzed simultaneously to urine samples (23 from immunocompetent hosts and 15 from kidney recipients). *E*. *coli* in urine samples was identified during routine microbiological diagnostics based on MALDI TOF spectrometry. (Microflex, Bruker, USA) Virulence factors were confirmed with polymerase chain reaction (PCR), and the following genes were included in the study: *fimH*, *papC*, *csgA*, *flu* (named further as Ag43), and *tosA*. Genomic bacterial DNA was isolated with GeneMATRIX Bacterial, and Yeasts Genomic DNA Purification Kit (EURx, Poland, cat. no. E3580-01), and the quality of isolated DNA was verified with NanoDrop 1000 Spectrophotometer (ThermoScientific, USA). Isolated DNA was stored at -20°C until further analyses. PCR reactions were performed on Applied Biosystem Veriti 96 Thermal Cycler (Upland, CA, USA). Specific primers and respective references are listed in Table 1 [15, 32, 33]. The primer for the c*sgA* gene was self-designed using the online tool Primer 3 [34]. *E*. *coli* strain CFT073 (ATCC 700928) was used as a control.

**Table 1:** List of primers used to adhesins genes identification.

Gene	Primer sequence 5’-3’	Product size bp	References
*fimH*	fimHF: TGCAGAACGGAAAGCCGTGG	508 bp	Johnson and Stell 2000 [32].
	fimHR: GCAGTCACCTGCCCTCCGGTA		
*papC*	papCF: GTGGCAGTATGAGTAATGACCGTTA	200 bp	Johnson and Stell 2000 [32].
	papCR: ATATCCTTTCTGCAGGGATGCAATA		
*flu*	fluF: ACGCACAACCATCAATAAAA	600 bp	Mendez-Arancibia *et al*. 2008 [33].
	fluR: CCGCCTCCGATACTGAATGC		
*csgA*	csgAF: CTCTGGCAGGTGTTGTTCCT	152 bp	This study [34]
	csgAR: ATCAGAGTTACGGGCGTCAG		
*tosA*	tosAF: GCACAGCATAACGGGAAAAT	589 bp	Xicohtencatl-Cortes *et al*. 2019 [15].
	tosAR: CCAGCATGTTACCACGAATG		

### Computational analysis

Both statistical and data mining algorithms were applied in the analysis. Shapiro-Wilk test was used to verify the distribution of the variables. The ANOVA test was used to compare variables between kidney recipients and two other groups. The frequency of features in each group was verified with the chi-square test. Decision tree models were used to assess the hierarchy of individual factors. The principles of decision tree modeling were described elsewhere [8, 35, 36]. Briefly, these algorithms represent supervised machine learning methods. They are applied to extinguish highly homogenous subgroups (leaves) identified with a class label and characterized by a set of attributes. The attributes are tested one by one in recurrent operations to assess their classification potential and eventually present these in the hierarchical structure. Consequently, decision trees are valued for their ability to present a systemic classification of objects characterized by a set of attributes and a class label.

Three evaluation rates are used to assess the performance of the models: overall accuracy, precision, and recall. Precision identifies how many positive objects are truly positive, while recall verifies how efficient the algorithm is in recognizing a positive object in general. This assessment is strengthened with a cross-validation algorithm in which the model is trained on one randomly selected subset of data and tested on the other. This operation is repeated in a manner dependent on algorithm settings, most preferably ten times. Statistica version 13.3 and RapidMiner Studio software were used in the analyses. The exact workflow has been detailed described in the *Results* section.

### Ethical issues

The study was approved by the Local Bioethical Committee at Pomeranian Medical University in Szczecin (decision number KB-0012/136/17) Verbal consent has been obtained from all participants.

## Results

### The prevalence of adhesins

The prevalence of genes for particular adhesive factors was following in the analyzed strains: *tosA* 20.5% *papC* 28.2%, *flu* (Ag43) 43.5%, *csgA* 94.8% *fimH* 97.4% (S1 Fig). Type I and P fimbriae appeared the most prevalent. There were no statistically significant differences in the prevalence of particular adhesins between kidney recipients and immunocompetent hosts.

### Comparison of interleukin urine concentrations regarding the immune status

**Fig 1:**
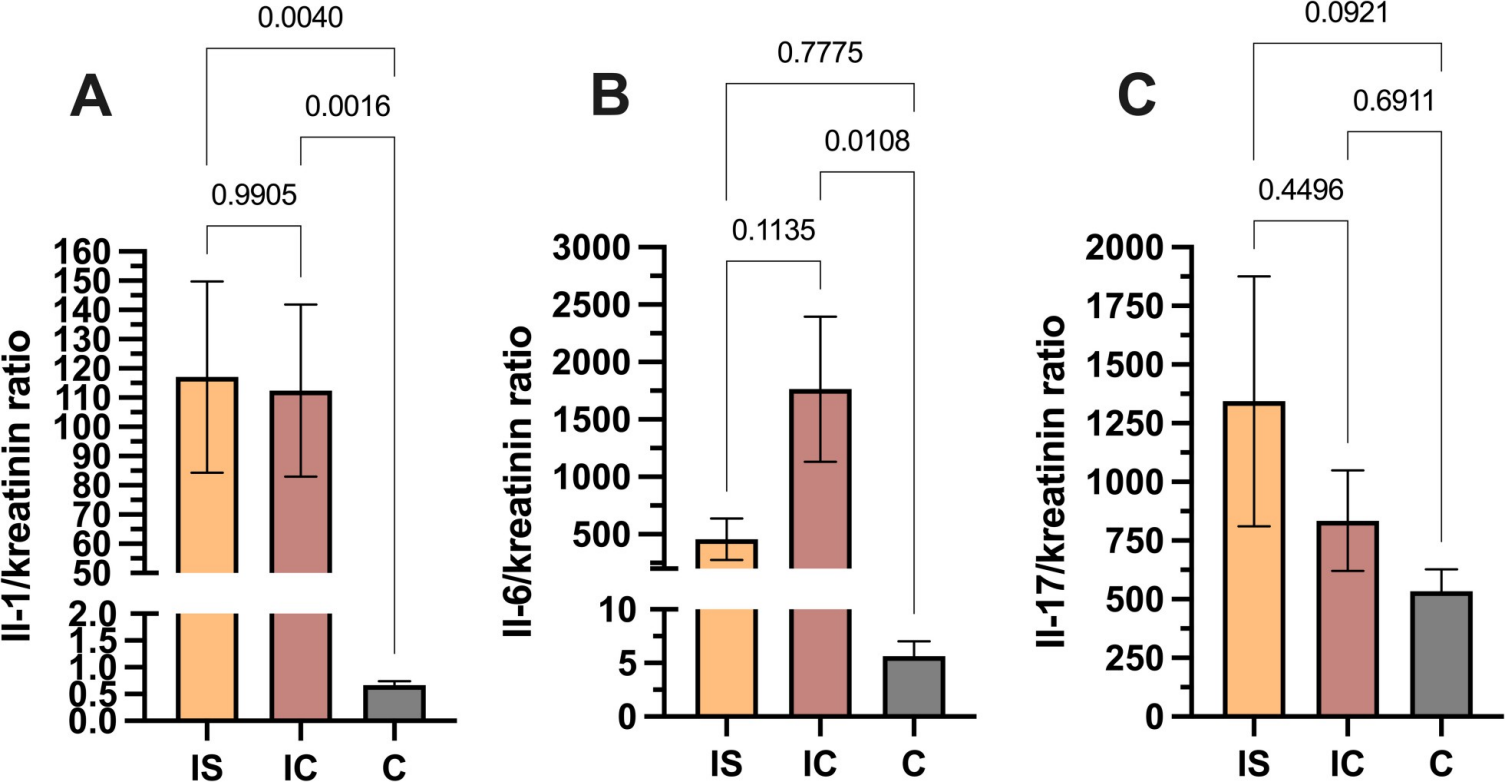
Differences in cytokine concentrations between kidney recipients (immunosuppressed—IS), immunocompetent UTI-affected hosts (IC), and controls (C). A displays a significant difference in IL1β concentrations between IS and IC and controls and no significant difference between IS and IC groups. B depicts a considerable difference in IL6 concentrations between IC and controls and no significant difference both between IS and IC as well as IS and controls. There is also low diversity in IL 6 urine concentrations in IS group. C displays no significant differences in IL 17 concentrations between any of the groups. However, there is an increased diversity in urine IL17 concentrations in IS group.

There was no significant difference between eGFR values in kidney recipients and UTI-affected immunocompetent hosts. There were also no significant differences in IL1β/creatinine, IL6/creatinine, and IL17/creatinine ratios between kidney recipients and immunocompetent hosts. Simultaneously IL1β/creatinine ratio and IL6/creatinine ratios were higher in kidney recipients than in controls, while IL6/creatinine ratio was higher only in immunocompetent UTI-suffering hosts than in controls. There was no difference in IL17/creatinine ratios between kidney recipients, immunocompetent hosts and controls. Increased variation of IL17/creatinine ratio values in kidney recipients comparing to other groups was observed. The results are presented in Fig 1.

### Decision trees classification regarding the host’s immune status and the pathogen’s genotype

Regarding the distribution of interleukin/creatinine ratios, in particular increased variation of IL17/creatinine ratios in kidney recipients, we have decided to implement modeling based on decision tree algorithms in order to assess the impact of adhesins and immune status simultaneously. The principles have been briefly described in the *Materials and methods* section. As we investigated the interleukin/creatinine levels, we used this variable as a label. Consequently, the immune status and the adhesin genes were used as the attributes. The adhesins genes were treated independently. Due to their high prevalence, *fimH* and *csgA* genes were excluded from the analysis. As we decided to implement a rather classification than regression models, we established two sets of classes: low and high interleukin/creatinine ratios for each interleukin, respectively. The median value of each interleukin/creatinine ratio was used as the borderline for this distinction so that the numbers of both classes were approximately equal in each group. Median values themselves as well as the values below level of detection were incorporated into low ratios subsets. Subsequently, we built two classification models for high and low interleukin/ratios separately. What needs emphasizing is that, in terms of the algorithms, we treated single interleukin assessments as observations, but not the individuals themselves. As a result, we analyzed all the classes of high and low ratios independently. Consequently, the total number of observations was 99 for *high* and 102 for *low ratios*, respectively. We applied a cross-validation algorithm to establish each model’s performance parameters with ten automatic training and testing folds.

**Fig 2:**
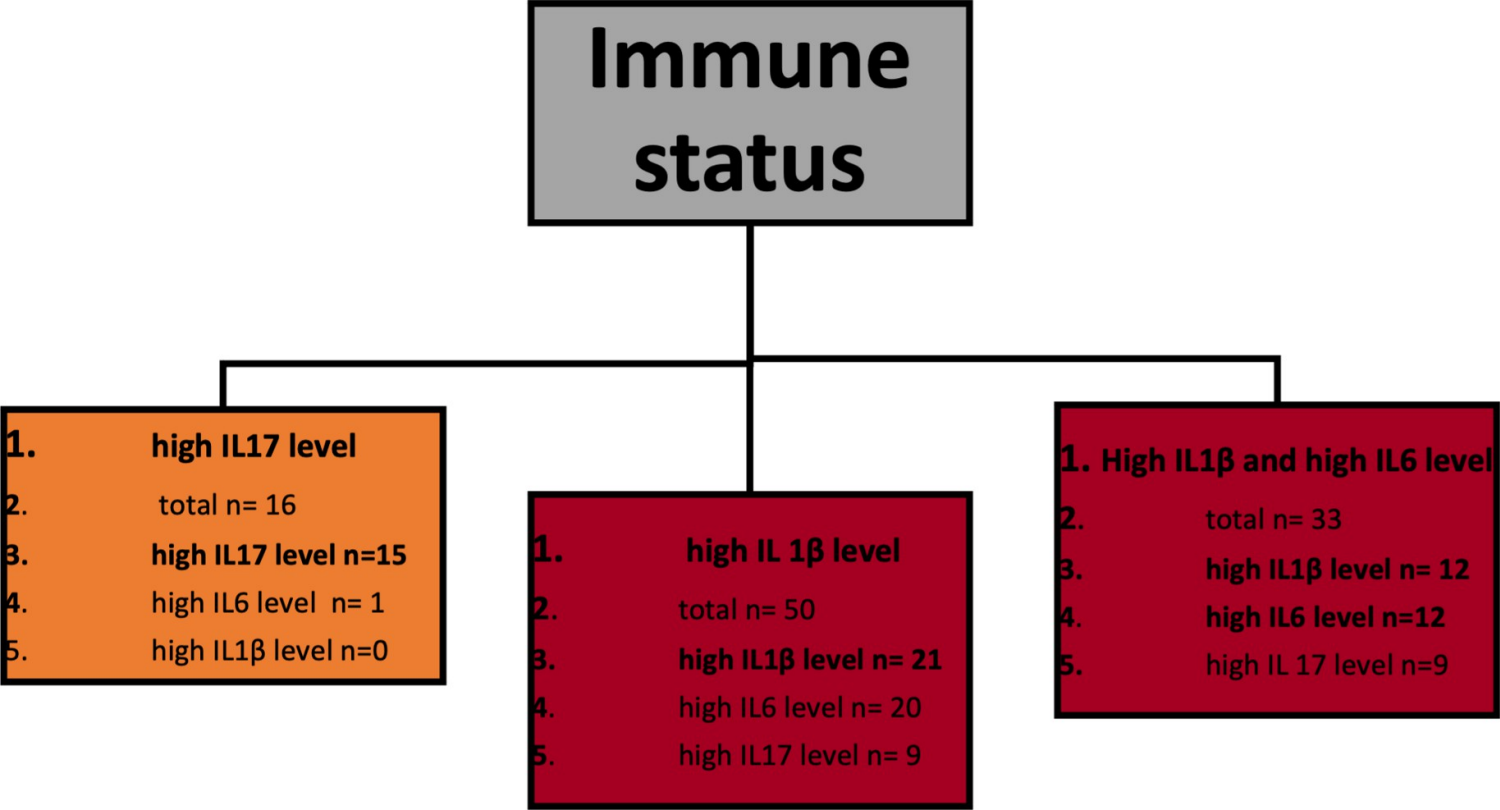
Classification model of high urine interleukin levels. This model classifies three separate labels: *high IL17 level* vs. *high IL1β level* vs. *high IL6 level*. All three labels have been treated independently. Each of the leaves contains following information: point 1 –dominating label presented in bold, point 2- n- total number of observations classified in this leaf, points 3–5: the numbers of each observations classified in this leaf including the dominating one.

**Fig 3:**
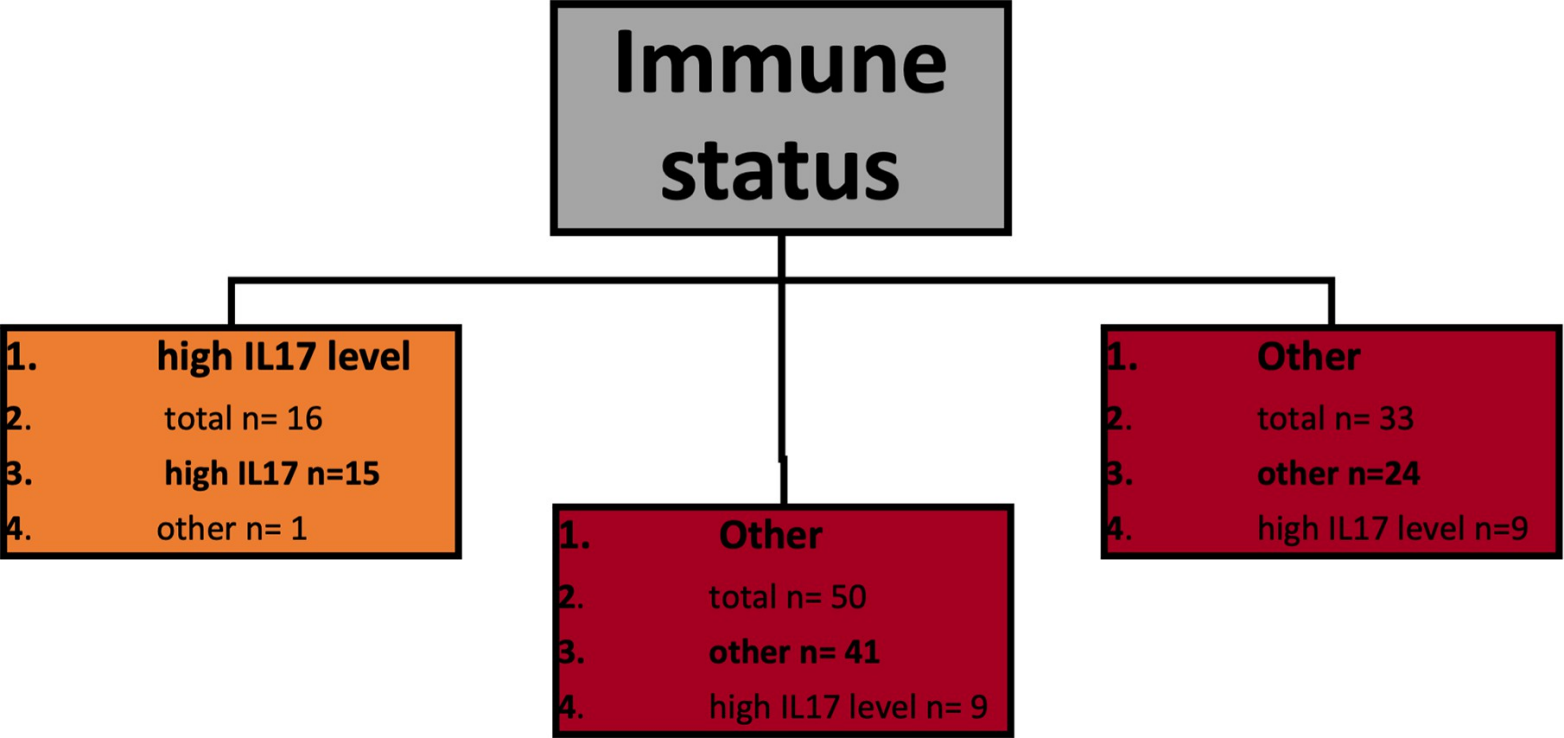
Classification model of high urine interleukin levels. This model classifies two separate labels: *high IL17 level* vs. *other*. In the opposite to Fig 2, in this model, labels *high IL1β* and *high IL6 level* have been consolided into one label *other*. Each of the leaves contains the following information: point 1 –dominating label presented in bold, point 2- n- total number of observations classified in this leaf, points 3–5: the numbers of each observations classified in this leaf including the dominating one.

Fig 2 presents a classification decision tree model in which high urine interleukin/creatinine ratios were used as labels and named as *high IL level*. Immune status appears the primary classification attribute, and a homogenous leaf that consists of *high IL17 level* controls has been extinguished. Kidney recipients appear as a heterogeneous group regarding the content of *high IL levels* samples. The overall accuracy of this model is relatively low (39.33%). However, the precision rate for *high IL17 level* classification is outstanding (93.75%). (Table 2) As the number of samples labeled as high *IL1β level* and *high IL6 level* was approximately equal in *kidney recipients* and *immunocompetent hosts* leaves, we repeated this classification using *high IL1β level* and *high IL6 level* as one label named *other*. This model’s accuracy appears increased—over 80%, and so arises the class precision for *high IL17 level—*similarly to the previous model 93.75%—Fig 3 and Table 3. Apart from classification models performance, these outcomes were further proceeded as a classical statistic hypothesis. The prevalence of *high IL17 level* samples appeared significantly higher in controls (p = 0.00001) and not substantially different between kidney recipients and UTI-affected immunocompetent hosts (p = 0.3).

**Table 2:** Performance parameters of classification model targeting three labels *high IL17 level* vs. *high IL1β level* vs. *high IL6 level* independently. Accuracy 39.33%.

	True *high IL6 level*	True *high IL17 level*	True *high IL1β level*	Class precision
**Predicted *high IL 6 level***	11	11	20	26.19%
**Predicted *high IL 17 level***	1	15	0	**93.75%**
**Predicted *high IL 1β level***	21	7	13	31.71%
**Class recall**	33.33%	45.45%	39.39%	

**Table 3:** Performance parameters of classification model targeting binary labeling *high IL17 level* vs. *other*. Accuracy **80.89%**.

	True *other*	True *high IL17 level*	Class precision
**Predicted *other***	65	18	78.31%
**Predicted *high IL17 level***	1	15	**93.75%**
**Class recall**	98.48%	45.45%	

**Fig 4:**
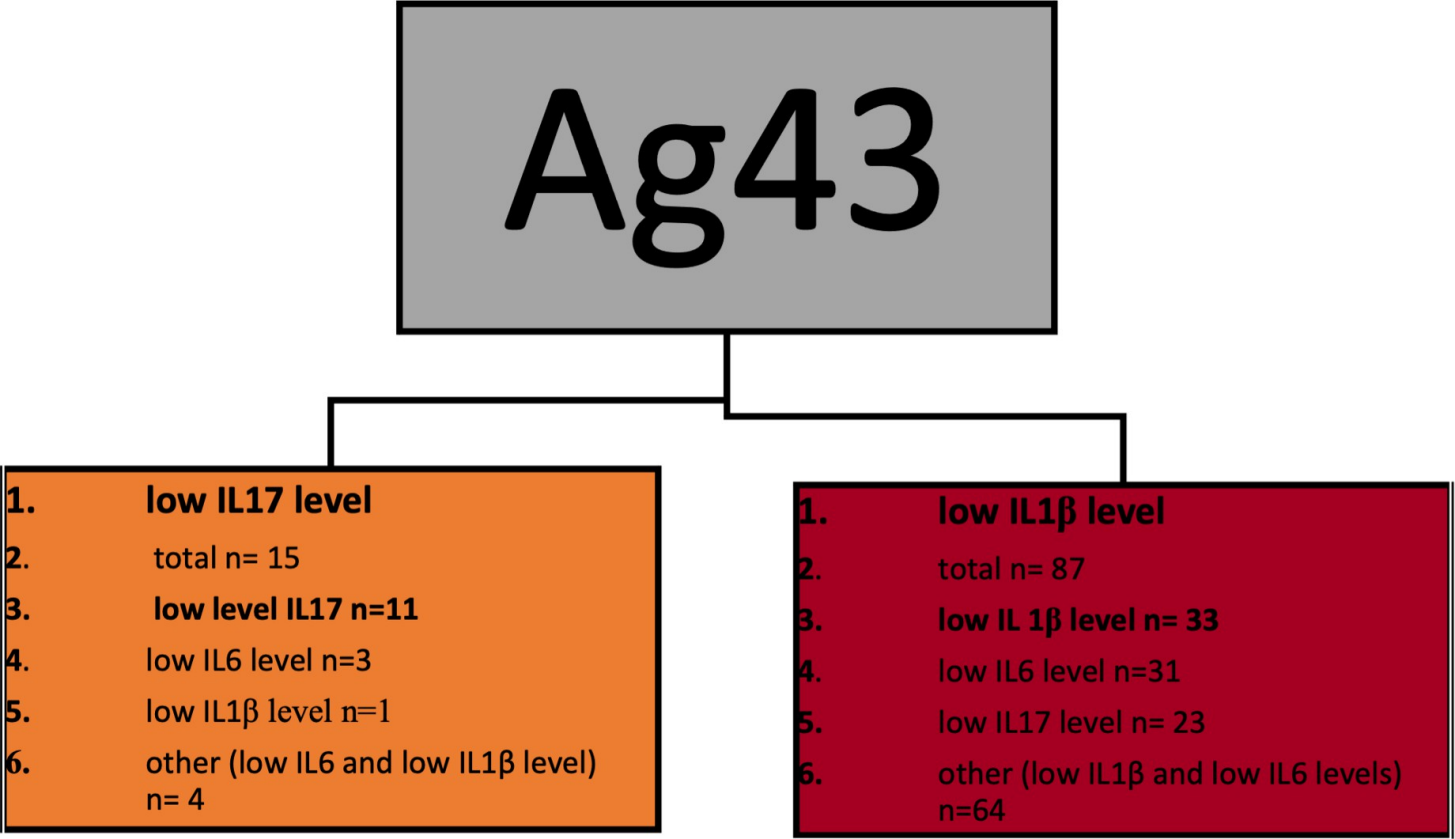
Classification model of low interleukin levels. This model targeted three separate labels: *Low IL17 level* vs. *low IL1β level* vs. *low IL6 level*. However, the consolided information about labels other than *low IL17* have also been presented. Each of the leaves contains the following data: point 1 –dominating label presented in bold, point 2- n- total number of observations classified in this leaf, points 3–5: the numbers of each observations classified in this leaf including the dominating one, point 6- the number of observations other than *low Il17 (low IL1β* and *low IL6*) taken together.

Fig 4 presents a classification model in which low interleukin/creatinine ratios were used as labels and named as *low IL level*. The presence of the Ag43 gene in a sample appears as the only classification attribute regardless of an individual’s immune status and the presence of genes for other adhesins. Simultaenously to the previous model, the outcomes were proceeded as statistical hypothesis and *Low IL17 level* samples appeared significantly more prevalent in Ag43-positive leaf (p = 0.00001). The overall accuracy of this model is limited. Nevertheless, the precision for *low IL17 level* classification remains outstanding (Table 4). Like the first one, the accuracy was improved once the model was turned into binary–labeled—*low IL17 levels* vs. *others* (Table 5).

**Table 4:** Performance parameters of classification model targeting three labels *low IL6 level* vs. *low IL17 level* vs. *low IL1β level* independently. Accuracy 38.27%.

	True *low IL6 level*	True *low IL17 level*	True *low IL1β level*	Class precision
**Predicted *low IL6 level***	2	3	4	22.22%
**Predicted *low IL17 level***	4	11	4	**57.89%**
**Predicted *low IL1β level***	28	20	26	35.14%
**Class recall**	5.88%	32.35%	76.47%	

**Table 5:** Performance parameters of classification model targeting binary labeling *low IL17 level* vs. *other*. Accuracy **71.64%**.

	True *low IL17 level*	True *other*	Class precision
**Predicted *low IL17 level***	23	62	**64.71%**
**Predicted *other***	11	6	72.94%
**Class recall**	32.35%	91.18%	

### Comparison of IL17/creatinine ratio regarding Ag43 presence

**Fig 5:**
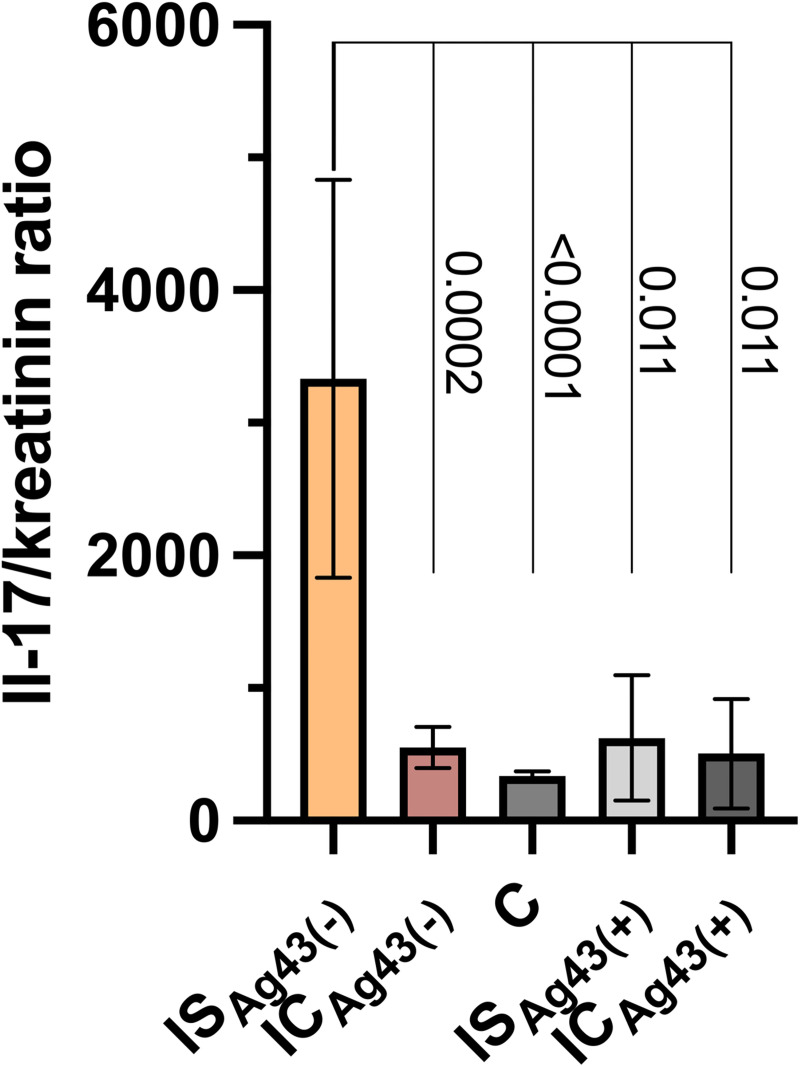
Differences in urine IL17 concentration between kidney recipients (IS), immunocompetent hosts (IC), and controls (C) regarding the presence of Ag43 gene in the isolated UPEC strain. Significantly higher values were observed in urines from kidney recipients infected with Ag43-negative strains. The differences between other groups are not significant.

According to classification tree findings, the workflow was completed with statistical analysis of IL17/creatinine ratios in Ag43 positive and Ag43 negative groups. IL17 concentration was significantly higher in urines obtained from kidney recipients infected with Ag43-negative UPEC strains. The results are presented in Fig 5.

## Discussion

This study aimed to compare the inflammatory response associated with UPEC-related urinary tract infection in kidney recipients and immunocompetent hosts regarding adhesins repertoire present in the pathogens. The quality of inflammation was characterized by IL1β, IL6, and IL17 urine levels, respectively. Healthy controls were included in the study. Hypothesis- driven statistical analysis and machine learning classification models were implemented subsequently. There was no significant difference between each interleukin’s concentrations in kidney recipients and immunocompetent hosts suffering from UTI in the classical statistical analysis. However, IL1β concentrations appeared higher in kidney recipients than in controls.

In contrast, IL17 concentrations appeared similar in all groups: kidney recipients, immunocompetent hosts and controls. Highly differentiated IL 17 concentrations in kidney recipient’s urine are worth noting. In the machine learning analysis, IL17 concentrations above median appeared more prevalent in samples from controls while IL17 concentrations below median, in urines of patients infected by Ag43 gene-positive UPEC strains, regardless of the host’s immune status. In a subsequent hypothesis-driven statistics analysis, higher IL17 levels appeared in urines from kidney recipients infected with Ag43- negative UPEC. The findings from machine learning models and statistical analysis correspond to each other.

IL1β represents the proinflammatory mediator released by innate immunity cells when activated by lipopolysaccharide of *E*. *coli*. The concentrations of IL1β did not differ between kidney recipients and immunocompetent hosts in our study. Increased urine IL1β concentrations during the UTI episode were discovered by Sundac *et al*. [37]. Similarly, Gadalla *et al*. confirmed the association between increased IL1β urine levels and UTI using machine learning algorithms [38]. Furthermore, Sheu *et al*. indicated higher urine IL1β levels in children with general symptoms accompanying UTI and renal scarring confirmed in imaging diagnostics [39]. However, all the cited studies address immunocompetent hosts, and comparative research on kidney recipients is limited in the context of UTI. Nevertheless, experimental models of pyelonephritis indicate more complex properties of IL1β. Knockout mice without the IL1β gene were found more susceptible to inflammatory damage to the kidney when compared with wild-type mice. Apart from pro-inflammatory properties, IL1β was also found to induce immunomodulatory cytokines such as IL10 and IL4 [40].

At the same time, IL6 has been considered the most sensitive inflammatory marker [41]. It has also been recognized to be induced both by lipopolysaccharide and P fimbriae [42]. The protective role of IL6 during specifically UPEC-related UTI has been proven. Similarly, in the pyelonephritis mice model, higher mortality in IL6 knockout mice was found when infected with *E*. *coli* CFT073 strain [43]. Saghedi *et al*. have also observed increased IL6 urine concentration both during UTI and asymptomatic bacteriuria [44]. Our study found no statistical difference in IL6 urine concentration between kidney recipients and immunocompetent hosts. However, concerning Fig 1, this similarity appears less evident than in terms of IL1β. There is a decreasing trend in IL6 concentration when considered immunocompetent hosts, kidney recipients, and controls. The difference between kidney recipients and immunocompetent hosts could possibly achieve statistical significance when analyzed on a larger population, which points to the limitation of our study. A slight variation of IL6 levels in samples from kidney recipients is remarkable.

Nevertheless, both IL1β and IL 6 have been found correlated with kidney allograft injury [24, 27, 45]. Our study found increased urine concentrations of IL1β but not IL6 in kidney recipients compared to controls. The connection between UTI episodes and kidney allograft injury has not been fully clarified. Although previously such has not been strongly considered, more recent data indicates the negative influence of UTI on long-term allograft function [46]. Investigating the relation between IL1 and IL6 and kidney injury based on a single time point measurement during the UTI episode is challenging and was not aimed at this study. However, in our opinion, the follow-up of cytokine dynamics after UTI episodes in immunocompetent hosts and kidney recipients deserves to be considered.

No significant difference was found in IL17 urine concentration either between kidney recipients and controls or between kidney recipients and immunocompetent UTI-affected patients. In the aforementioned Sundac *et al*. findings, urine IL17 concentration was elevated in UTI episodes. However, as mentioned, this study did not investigate immunocompromised hosts [37]. Animal models indicate the role of IL17 during UTI, derived mainly from Tγδ cells [23, 47]. To evaluate the interfering part of immune status and pathogens virulence, we implemented decision tree modeling and classified the individuals in each group as high and low IL concentrations. All the models represented the highest precision in organizing regarding IL17 concentration, either high or low. As mentioned in the *Results* section, the models’ overall accuracy was limited when classifying three labels representing three cytokines’ concentrations separately and increased consequently to restrict the number of the labels to two. However, this refers only to the IL 17 classification in which the precision rate was outstanding regardless of the accuracy of the model. We performed this operation on two other cytokines using the labels *high/low IL 1β* vs. *other* and *high/low IL6* vs. *other*, respectively. As a result, each model’s accuracy did not show an increase, and the models appeared entirely inefficient in classifying IL 1β and IL 6 concentrations (S1 File). Taking all the above under the circumstances, we conclude that our models were the most efficient in classifying IL 17 concentrations.

Interestingly high IL17 concentrations appeared the most prevalent in the control group. It is even more evident when considered higher urine creatinine concentrations indicated by higher eGFR in this group. Regarding the role of Tγδ cells in local IL17 production, we hypothesize that IL17 plays a role of constantly present innate defense factor. Simultaneously, low IL17 concentrations were more prevalent in urines infected with Ag43 positive UPEC, but higher IL17 levels were present in kidney recipients infected with Ag43-negative UPEC. Hence, our study has indicated the interplay between host and pathogen-derived factors in shaping local immunity. Despite existing knowledge about adhesins immunogenicity, discussing this issue appears challenging. The amount of data on how the adhesins other than fimbriae shape the cytokines responses is minimal. Moreover, fimbriae appear the most frequent adhesins in UPEC, so that are inefficient as classifiers. To our best knowledge, this is the first study indicating the connection between Ag43 in UPEC, immune status of the host, and urine IL17 concentration. What gains attention is the role of Ag43 in biofilm formation [11, 16, 17]. We hypothesize that once the biofilm is recognized as a bacterial evasion mechanism, this can also shape local IL17 production, as also hypothesized, constantly present in the urinary tract.

This study represents several limitations. The number of observations may have biased verifying the difference in IL6 and IL 17 concentrations between kidney recipients and immunocompromised hosts. Cross-validation and non-trivial approach to data used in machine learning algorithms made the data more easily approachable despite sample limitations. It complies with the general rules of using machine learning [30, 35] and corresponds with the general idea of using data mining to find non-trivial associations within the data. Although, in our decision trees modeling, we excluded age and gender as these did not represent independent variables—the age highly associated with controls and female gender with kidney recipient’s status. Despite our finding on the role of Ag43 in kidney recipients and the UTI, highly differentiated levels of IL17 in kidney recipients’ urine samples remain unresolved. We, therefore, hypothesize a set of other underlying host and pathogen-dependent factors that can classify kidney recipients into different subgroups regarding urine IL17 level during a UTI episode. At the host’s side immunosuppressive protocols need further investigation on this area. At the pathogen’s side, with regard to the results of this study, Ag43 adhesin should be primarily considered. Machine learning algorithms represent the future direction in such investigations. Eventually, as mentioned previously, we also find it considerable to follow-up IL17 and other cytokine levels when concerning the impact of UTI on kidney allograft function.

## Conclusions

The combination of the immune status of an individual and Ag43 status of UPEC strain determined urine IL17 level in the analyzed group. Ag43 gene presence was associated with lower IL 17 levels. IL17 levels above median were more prevalent in controls, although the difference in average IL17 levels was not statistically significant between all groups. IL1β and IL6 levels did not differ between kidney recipients and immunocompetent host during the UTI episode.

## Supporting information

S1 Fig The prevalence of adhesins genes. (TIFF)

S1 File Other models precision and accuracy. (DOCX)

S2 File Data. (XLSX)
