# Supplementary figures and images for: The role of uropathogenic Escherichia coli adhesive molecules in inflammatory response- comparative study on immunocompetent hosts and kidney recipients
Source: PLoS One. 2022 May 23;17(5):e0268243. doi: 10.1371/journal.pone.0268243 (PMC9126363; doi:10.1371/journal.pone.0268243)

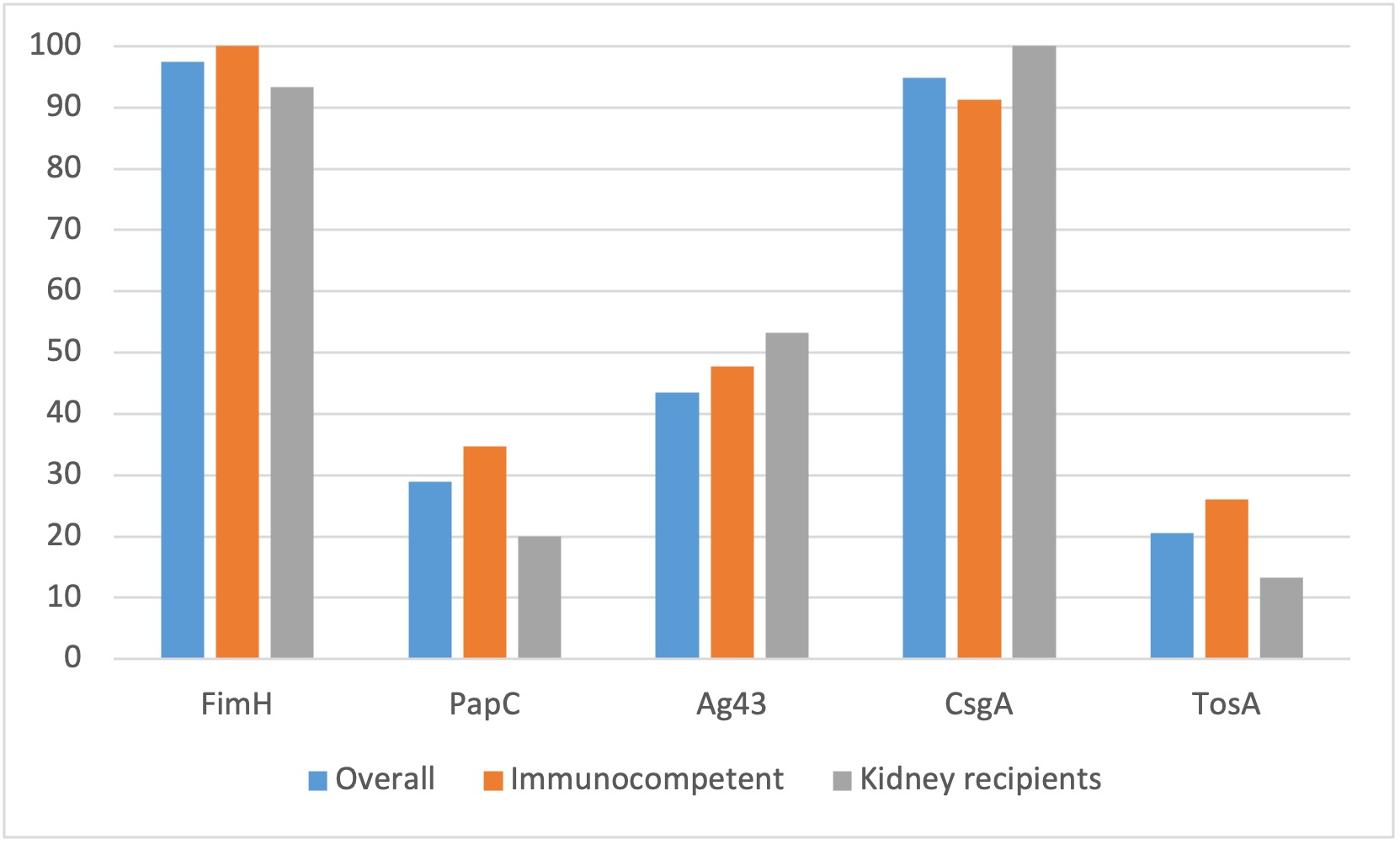

Supplement: S1 Fig — (TIFF) [file pone.0268243.s001.tiff]
