# Supplementary material for: The role of uropathogenic Escherichia coli adhesive molecules in inflammatory response- comparative study on immunocompetent hosts and kidney recipients
Source: PLoS One. 2022 May 23;17(5):e0268243. doi: 10.1371/journal.pone.0268243 (PMC9126363; doi:10.1371/journal.pone.0268243)
Supplement: S1 File — (DOCX) [file pone.0268243.s002.docx]

Supplementary material 2.

Model accuracy and class precision for predicted *high IL1β* and *high IL6* labels vs. *other* as well as *low IL1β* and *low IL6* labels vs. *other*

| **Model** | **Model accuracy** | **Class precision** |
| --- | --- | --- |
| High IL1β vs. other | 63% | 20% |
| High IL6 vs. other | 66% | 0% |
| Low IL1 β vs. other | 66% | 0% |
| Low IL6 vs. other | 66% | 0% |
